# The *arouser* EPS8L3 Gene Is Critical for Normal Memory in *Drosophila*

**DOI:** 10.1371/journal.pone.0022867

**Published:** 2011-07-27

**Authors:** Holly LaFerriere, Daniela Ostrowski, Douglas J. Guarnieri, Troy Zars

**Affiliations:** 1 Division of Biological Sciences, University of Missouri, Columbia, Missouri, United States of America; 2 Department of Anatomy, University of California, San Francisco, San Francisco, California, United States of America; Alexander Flemming Biomedical Sciences Research Center, Greece

## Abstract

The genetic mechanisms that influence memory formation and sensitivity to the effects of ethanol on behavior in *Drosophila* have some common elements. So far, these have centered on the cAMP/PKA signaling pathway, *synapsin* and *fas2-*dependent processes, *pumilio*-dependent regulators of translation, and a few other genes. However, there are several genes that are important for one or the other behaviors, suggesting that there is an incomplete overlap in the mechanisms that support memory and ethanol sensitive behaviors. The basis for this overlap is far from understood. We therefore examined memory in *arouser* (*aru*) mutant flies, which have recently been identified as having ethanol sensitivity deficits. The *aru* mutant flies showed memory deficits in both short-term place memory and olfactory memory tests. Flies with a revertant *aru* allele had wild-type levels of memory performance, arguing that the *aru* gene, encoding an EPS8L3 product, has a role in *Drosophila* memory formation. Furthermore, and interestingly, flies with the *aru^8–128^* insertion allele had deficits in only one of two genetic backgrounds in place and olfactory memory tests. Flies with an *aru* imprecise excision allele had deficits in tests of olfactory memory. Quantitative measurements of *aru* EPS8L3 mRNA expression levels correlate decreased expression with deficits in olfactory memory while over expression is correlated with place memory deficits. Thus, mutations of the *aru* EPS8L3 gene interact with the alleles of a particular genetic background to regulate *arouser* expression and reveals a role of this gene in memory.

## Introduction

In *Drosophila*, the genetic basis for sensitivity to the effects of ethanol on behavior and memory formation have some common elements. Parts of the cAMP/PKA signaling cascade, as well as the Fas2 and synapsin proteins, have been implicated in both behaviors [1]–[3]. Furthermore, tests of memory mutants in ethanol sensitivity and new ethanol sensitive mutants in memory led to the conclusion that there are several processes important for both behaviors (e.g., *pumilio*-based regulation of translation) [4], [5]. Although there seems to be an over-representation of genes important for both memory formation and ethanol sensitivity, a direct test of the correlation between ethanol sensitivity and memory with over fifty different mutant lines failed to find a significant correlation [4], [5]. Together, these results suggest that there are both differences and commonalities in the genes important for both behaviors, and with enough information one should be able to understand the molecular and cellular bases for the common mechanisms.

The EPS8 family of proteins have been shown to be important regulators of behavior in mouse and fly. In the mouse, knock-out of the EPS8 gene leads to a resistance to the sedation and locomotion effects of ethanol [6]. Furthermore, the cellular function of EPS8, regulating actin dynamics, suggests that ethanol effects on behavior are influenced by neuron remodeling. Indeed, actin remodeling within discrete regions of the brain could be important for regulating these effects. In *Drosophila*, the paralog EPS8L3 has been implicated in regulating the effects of ethanol on behavior [7].

Although dependent on a relatively small number of genes, there seems to be an over-representation of genes important for regulating both ethanol sensitivity and memory formation [4], [5]. Because of the *aru* EPS8L3 link with ethanol sensitivity, we asked whether *aru* EPS8L3 is necessary for proper memory performance in *Drosophila*. We examined the role of *aru* EPS8L3 in aversive short-term place memory and aversive olfactory memory. In place memory, flies are allowed to wander in a short narrow chamber, one half of which is associated with a high non-preferred temperature [8], [9]. Providing the high-temperature contingency usually leads to avoidance of that chamber half, even when the danger of rising temperatures is removed. In classical olfactory conditioning, one of two odors is paired with electric shock [10], [11]. When given a choice between those two odors normal flies avoid the shock-associated odor. Flies from two different wild-type strains and three *aru* EPS8L3 alleles in both wild-type genetic backgrounds were tested in these two learning paradigms. Furthermore, the expression level of *aru* EPS8L3 was examined by quantitative real time (qRT) PCR in all strains. Our results indicate that expression levels of *aru* EPS8L3 have a significant effect on *Drosophila* memory performance.

## Results

**Figure 1:**
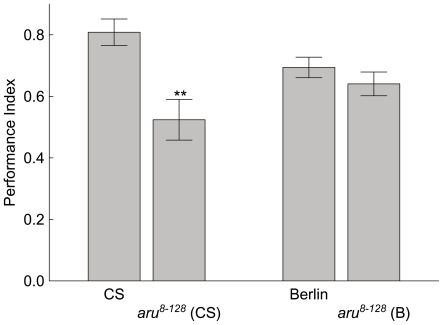
The *aru^8–128^* allele reduces place memory performance. Wild-type CS and Berlin (B) flies, as well as *aru^8–128^* flies in either the CS or B genetic backgrounds were trained for 20 min and then examined for place memory directly afterward. The mutant memory performance was statistically different from wild-type only in the CS genetic background (CS vs. *aru^8–128^* (CS), ** = p<0.01, N = 215; Berlin vs. *aru^8–128^* (B), p>0.1, N = 643). The values are means and error bars represent SEMs.

We tested the role of *aru* EPS8L3 in memory. The *aru^8–128^* allele was identified in a P-element insertion screen for ethanol sensitive mutants [4], [7]. Flies of this genotype were examined in two different genetic backgrounds, wild-type Canton S (CS) and Berlin. Our first experiments tested place memory in the heat-box. After conditioning for twenty minutes with 41°C negative reinforcement, place memory levels were reduced ∼30–35% in *aru^8–128^* flies compared to the CS wild-type flies' performance (Figure 1). The *aru^8–128^* flies in the Berlin genetic background had only a non-significant reduction in memory performance (Figure 1).

**Figure 2:**
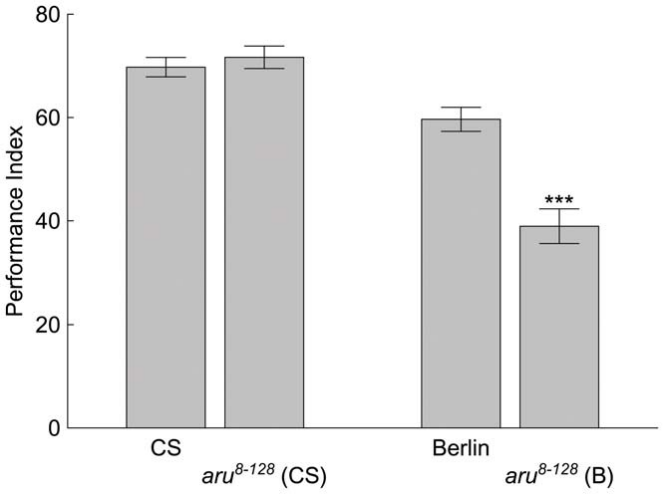
The *aru^8–128^* allele reduces olfactory memory performance. Wild-type CS and Berlin (B) flies, as well as *aru^8–128^* flies in either the CS or B genetic backgrounds were trained and tested for olfactory three minute memory. The memory performance was statistically different from wild-type only in the Berlin genetic background (CS vs. *aru^8–128^* (CS), F(1,10) = 0.44, p>0.1, N = 12; Berlin vs. *aru^8–128^* (B), F(1,12) = 27.5, *** = p<0.001, N = 14). The values are means and error bars represent SEMs.

We also determined whether *aru* EPS8L3 has a role in classically conditioned olfactory memory. The *aru^8–128^* flies had a reduced olfactory memory tested at three minutes following training in only the wild-type Berlin genetic background (Figure 2). The *aru^8–128^* flies in the Berlin background had an ∼40% reduction in memory compared to Berlin flies memory performance. Interestingly, *aru^8–128^* flies in the CS genetic background had memory levels that were similar to the CS flies performance levels. Thus, in olfactory classical conditioning, the *aru^8–128^* allele can influence memory levels, but this depends on the genetic background in which it is tested.

**Figure 3:**
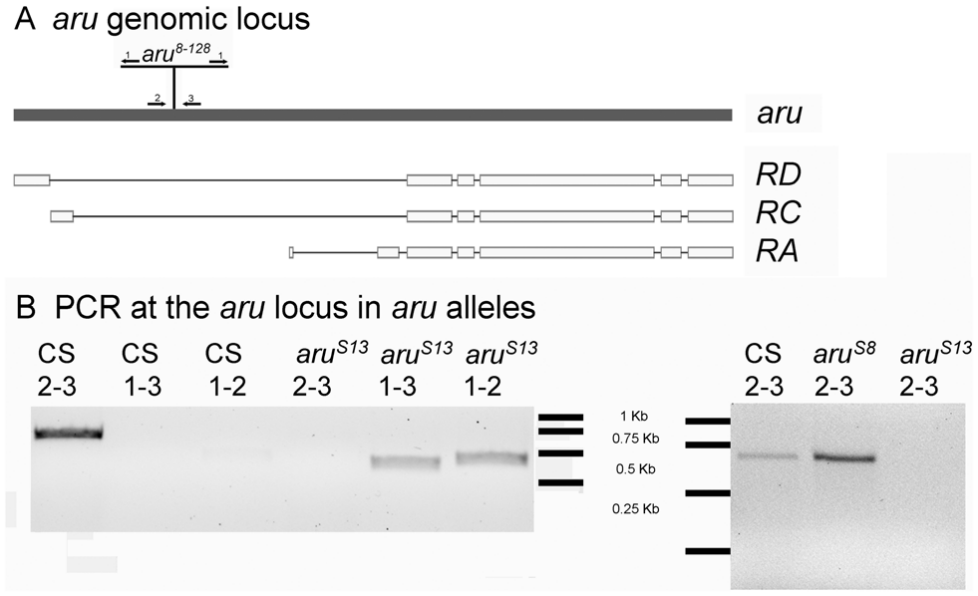
Molecular characterization of new *aru* alleles. A) The *aru^8–128^* allele is an insertion of a PGawB element in the genome corresponding to either the first intron of the RD and RC transcripts or 5′ of the RA transcripts. PCR primer pairs corresponding to the P-element inverted repeats (primer 1) and adjacent genomic sequence (primers 2 and 3) were used to characterize two new *aru* alleles, *aru^S8^*and *aru^S13^* (which were generated by re-mobilizing PGawB element in *aru^8–128^*flies). B) Amplification across the PGawB insertion site using primers 2 and 3 was possible from wild-type CS and *aru^S8^*, but not *aru^S13^*, genomic DNA. Amplification with the 1–2 and 1–3 primer pairs amplified the expected size products from genomic DNA from *aru^S13^* flies.

To better characterize the role of *aru* EPS8L3 in memory formation (Figure 3), the *aru^8–128^* insertion was remobilized. Two additional alleles of *aru* EPS8L3 have been generated, including a precise and an imprecise excision allele of the *aru^8–128^* insertion. Using PCR with oligonucleotide primers that anneal to the inverted repeats of the P-element and adjacent genomic DNA, we found that the *aru^S13^* allele still has parts of the P-element inserted in the genome. Amplification across the P-element insertion site failed from genomic DNA collected from *aru^S13^* flies, suggesting the P-element is still sufficiently large to prevent efficient amplification across the element. The *aru^S13^* allele, however, has lost significant components of the mini-*white* gene as the transgene does not complement a white-eyed phenotype when tested with the X-linked *w^1118^* allele. A second allele, *aru^S8^*, is a precise excision allele because the amplification product using oligonucleotide primers 2 and 3 (Figure 3) provide the same sized PCR product as that found using wild-type DNA as a template. Thus, the *aru^S13^*and *aru^S8^* alleles provide additional genetic tools for examining the role of *aru* EPS8L3 in memory.

**Figure 4:**
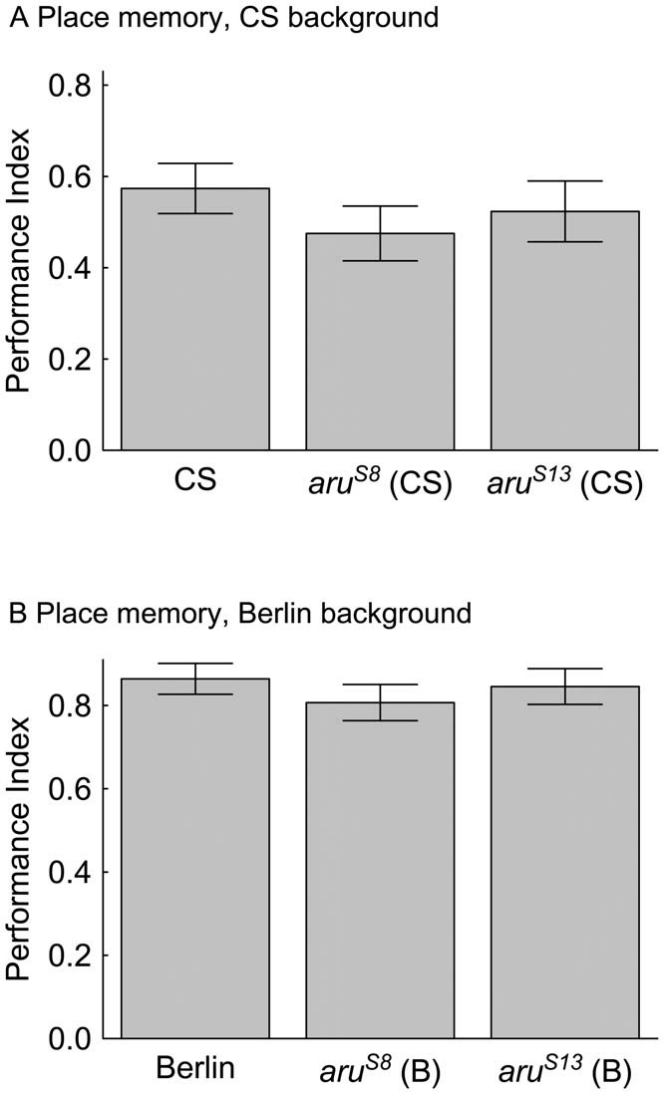
Place memory phenotypes of *aru^8–128^*, *aru^S8^*, and *aru^S13^* flies. Wild-type CS and Berlin, as well as flies with a precise excision (*aru^S8^*) and imprecise excision (*aru^S13^*) in both genetic backgrounds, were trained in the heat-box and tested for place memory. A) The memory score of flies from wild-type, *aru^S8^* and *aru^S13^*genotypes are presented, where there were no statistically significant differences detected in any of the genotypes (CS with *aru^S8^* (CS), and *aru^S13^* (CS) p's>0.1, N = 371). B) Flies with the *aru^S8^* and *aru^S13^*alleles in the wild-type Berlin background were also not significantly different (p's>0.1, N = 341). The values are means and error bars represent SEMs.

Flies with the *aru^S13^* and *aru^S8^* alleles in two different genetic backgrounds were examined in memory tasks. In place memory, flies with either the *aru^S8^* or *aru^S13^* alleles had memory levels that were statistically indistinguishable from the wild-type CS control levels (Figure 4A). Flies with either the *aru^S8^* or *aru^S13^* alleles in the Berlin background were also similar to wild-type Berlin memory levels (Figure 4B). The reversion of the *aru^8–128^* place memory phenotype with the precise excision allele (*aru^S8^*) in the CS background argues that the P-element insertion at the *aru* EPS8L3 locus causes the memory phenotype. In this paradigm, the *aru^S13^* allele in the CS background also reverts the phenotype to normal, suggesting that it is a less severe allele than *aru^8–128^*for this specific case.

**Figure 5:**
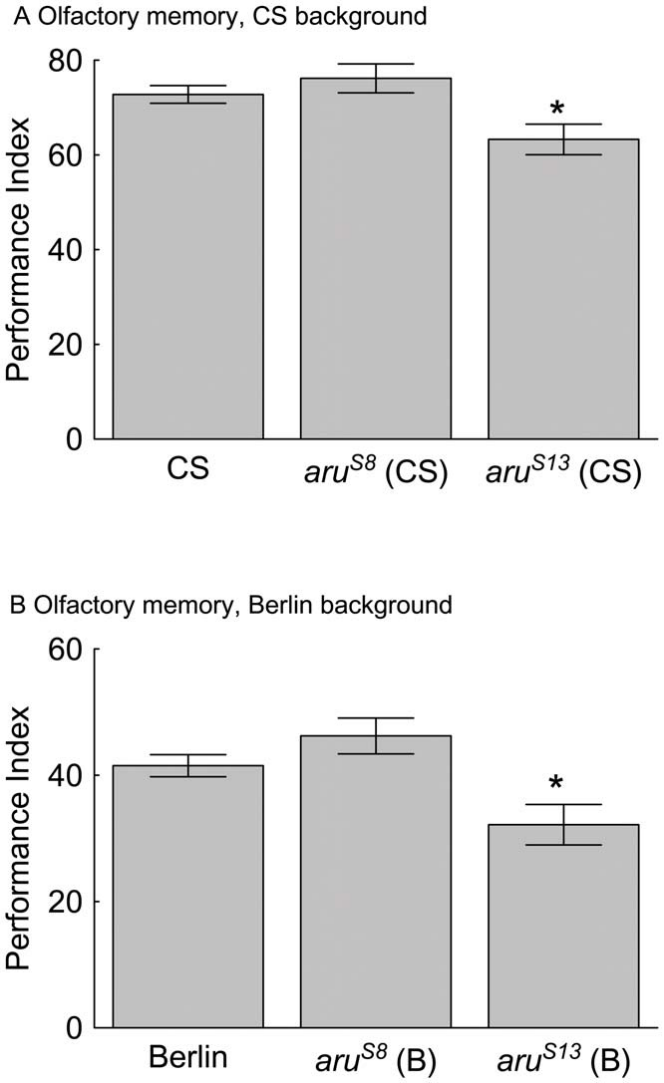
The olfactory short-memory defect of *aru^8–128^* flies is reverted to normal in flies with a precise excision allele (*aru^S8^*) but reduced in flies with an imprecise excision allele (*aru^S13^*). Wild-type CS and Berlin flies, as well as flies with a precise excision (*aru^S8^*) and imprecise excision (*aru^S13^*), were trained and tested for olfactory three minute memory. The short-term memory score of flies from wild-type, *aru^S8^* and *aru^S13^*genotypes are presented. A) The memory performance was statistically different in flies with the imprecise excision allele and their corresponding wild-type strain (CS with *aru^S8^* (CS) and *aru^S13^* (CS) F(2,15) = 5.8, p<0.01, * = p<0.05 with a Newman-Keuls *post-hoc* test of CS and *aru^S8^* (CS) with *aru^S13^* (CS), N = 18). B) Differences were also identified in flies from the wild-type Berlin backgrounds (Berlin with *aru^S8^* (B) and *aru^S13^* (B) F(2,29) = 7.3, p<0.002, * = p<0.05 with a Newman-Keuls *post-hoc* test of Berlin and *aru^S8^* (B) with *aru^S13^* (B), N = 32). The values are means and error bars represent SEMs.

Flies with the precise and imprecise *aru* EPS8L3 alleles were also tested for olfactory memory. The *aru^S8^* flies in the CS background are similar to the CS flies memory performance levels (Figure 5A). Olfactory memory is reverted to normal in flies with the *aru^S8^* allele in the wild-type Berlin background (Figure 5B). Interestingly, flies with the *aru^S13^* alleles perform at statistically lower levels than flies from either wild-type strain (Figure 5A and 5B). Thus, while the insertion allele of *aru^8–128^* in the CS background does not have an olfactory memory deficit, incomplete excision of the P-element results in lowered olfactory memory in this genetic background. The imprecise excision of the *aru^8–128^* P-element in the Berlin background still provides an olfactory memory deficit.

**Figure 6:**
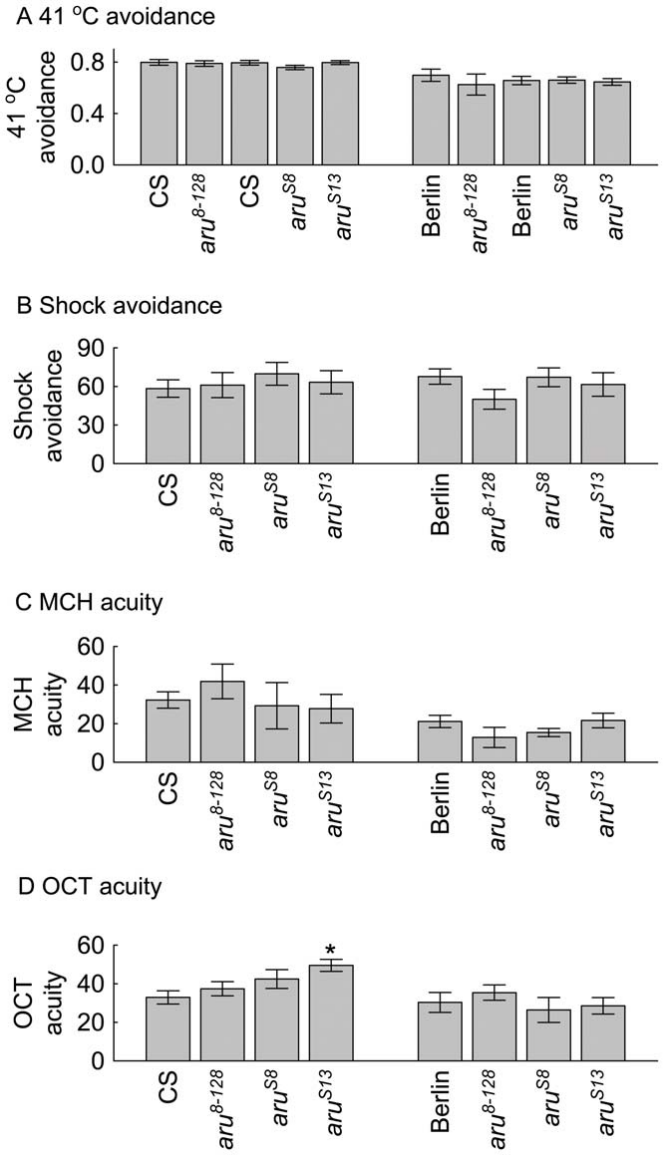
Control behaviors in wild-type CS, Berlin, and different *aru* EPS8L3 mutant flies. Control behaviors of wild-type and *aru* EPS8L3 mutant flies were largely similar. A) The avoidance of 41°C high temperature was similar between wild-type flies and all other flies with the three different *aru* EPS8L3 alleles (p's>0.1, N's between 100 and 240 for each genotype). B) Shock avoidance for flies with different *aru* EPS8L3 alleles were not statistically significantly different (CS compared to the three other *aru* EPS8L3 alleles: F(3,20) = 0.32, p>0.1; Berlin compared to the three other *aru* EPS8L3 alleles: F(3,24) = 1.29, p>0.1). C) Avoidance of MCH compared to ambient air was not statistically different between wild-type flies and flies with the three other *aru* EPS8L3 alleles (CS compared to the three other alleles: F(3,20) = 0.54, p>0.1; Berlin compared to the three other alleles: F(3,22) = 1.28, p>0.1). D) The only statistically significant difference in the different genotypes in the avoidance of octanol (OCT) was between flies from the CS and *aru^S13^* genotypes (CS background: F(3,20) = 3.5, p = 0.04, * = p<0.05 with Newman-Keuls *post-hoc* test with *aru^S13^* (CS) and CS; Berlin genetic background: F(3,20) = 0.57, p>0.1).

We examined control behaviors in flies with the different *aru* EPS8L3 alleles. We concentrated on testing naïve avoidance behaviors since these seem to be the most directly related to the memory paradigms, in contrast to some control-type experiments that others use. The latter, more complicated experiments, sometimes provide control-like results, but other times reveal novel behavioral phenomena [12]–[14]. The ability of *aru* EPS8L3 mutant flies to sense and avoid the odors, electric shock, and high temperatures was largely unaltered compared to wild-type flies (Figure 6). The only exception was the increased avoidance of the odorant 3-octanol by the *aru^S13^* flies compared to the wild-type CS flies levels. As the *aru^S13^* flies' olfactory memory levels were somewhat lower than wild-type CS levels, it seems likely that the increased sensitivity of the *aru^S13^* flies partially masks a stronger memory phenotype of these flies. However, it cannot be ruled out that the higher odor avoidance levels are partially responsible for the olfactory memory phenotype in this line. The other alleles tested in both genetic backgrounds suggest that the *aru* EPS8L3 memory phenotypes measured are independent of changes in the ability to sense and avoid the cues and reinforcing stimuli used in these paradigms.

**Figure 7:**
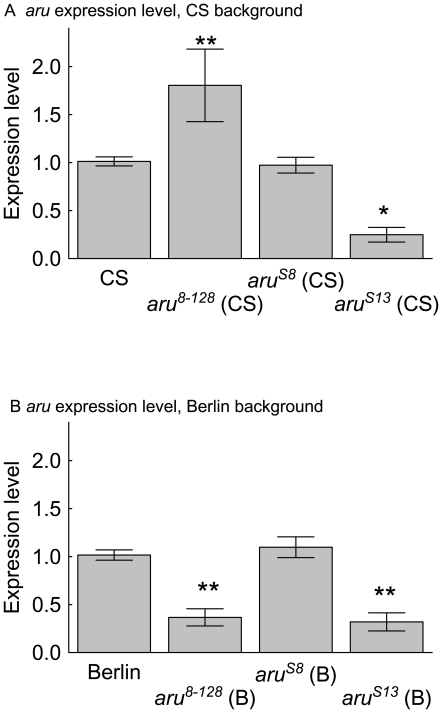
The relative expression levels of *aru* EPS8L3 is differentially altered by mutant alleles in different genetic backgrounds. A) In the wild-type CS background, flies with the *aru^8–128^* allele had significantly higher, while the *aru^S13^* flies had lower, *aru* EPS8L3 expression (F(3,41) = 8.48, p<0.0002. Newman-Keuls post-hoc test show differences between CS and both the *aru^8–128^* and *aru^S13^*alleles (P<0.05 = *, <0.01 = **)). B) In the wild-type Berlin background, flies with the *aru^8–128^* and *aru^S13^*alleles had significantly lower *aru* EPS8L3 expression (F(3,44) = 21.80, p<0.00001. Newman-Keuls post-hoc test show differences between Berlin and both the *aru^8–128^* and *aru^S13^* alleles (P's<0.01 = **).

We quantified *aru* EPS8L3 transcript levels by qRT-PCR in our first examination of the molecular mechanisms of how *aru* EPS8L3 mutation affects memory. Fly heads were used as a source for mRNA extraction, cDNA was synthesized, and qRT-PCR was performed on both *aru* EPS8L3 and *rp49* (as a control for mRNA levels in each extraction). In the CS genetic background, the *aru^8–128^* insertion allele had significantly higher levels of *aru* EPS8L3 expression compared to wild-type CS flies (Figure 7A). Flies with the molecular and behavioral revertant *aru^S8^* allele in the CS background had levels of *aru* EPS8L3 similar to wild-type CS flies, while the *aru^S13^* flies had a strongly reduced expression level for this gene. In the wild-type Berlin background, flies with either the insertion or imprecise revertant alleles (*aru^8–128^* and *aru^S13^*) had reduced *aru* EPS8L3 expression levels (Figure 7B). The molecular and behavioral revertant allele *aru^S8^* in the Berlin background had levels of *aru* EPS8L3 similar to that of the wild-type Berlin flies.

## Discussion

There are some genes that are both important for the behavioral responses to ethanol and memory formation. We add *aru* EPS8L3, with a significant reduction in memory levels in one of two genetic backgrounds to the short list of genes important for both behaviors. The genes that are now known to be critical for both types of behaviors center on regulators of the cAMP/PKA signaling pathway, *fasciculin2*, *synapsin*, *ethanol-sensitive* with *low memory* (*elm*), *aru* EPS8L3, and eleven mutants which also have long-term memory defects [1]–[5], [15]. The memory phenotypes we found with mutation of *aru* EPS8L3 adds to the conclusion that there are genetic subsystems that are critical for both ethanol sensitivity and memory.

The expression levels of *aru* EPS8L3 mRNA predict memory phenotypes. Examination of *aru* EPS8L3 mRNA levels from flies with wild-type, insertion, imprecise excision, and precise excision alleles showed that mRNA levels were either not altered, decreased, or increased. In all cases where there was a significant reduction in *aru* EPS8L3 mRNA levels olfactory memory levels, but not place memory levels, were reduced (Table 1). In contrast, only flies with the *aru^8–128^* insertion allele in the CS genetic background had an elevated level of *aru* EPS8L3 mRNA expression. This strain had the only significant effect on place memory, and no effect on olfactory memory. Future studies with over-expression of *aru* EPS8L3 will confirm these results, and may be used to determine where in the nervous system overexpression causes place memory decrements. Finally, the molecular revertant allele *aru^S8^* had normal levels of *aru* EPS8L3 mRNA expression and normal memory levels. Taken together, these results strongly argue that the changes at the *aru* EPS8L3 locus in the several mutant strains are the cause of the memory phenotypes measured.

**Table 1:** Relationship between *aru* EPS8L3 expression levels and memory.

Genotype	Placememory	Olfactory memory	*aru* EPS8L3 expression
CS	Normal	Normal	Normal
*aru^8–128^* (CS)	*Low*	Normal	*High*
*aru^S8^* (CS)	Normal	Normal	Normal
*aru^S13^* (CS)	Normal	**Low**	**Low**
Berlin	Normal	Normal	Normal
*aru^8–128^* (B)	Normal	**Low**	**Low**
*aru^S8^* (B)	Normal	Normal	Normal
*aru^S13^* (B)	Normal	**Low**	**Low**

*aru* EPS8L3 expression was either high (italics) or low (bold) depending on the mutant allele and genetic background (CS or B). Place memory was low (italics) when *aru* EPS8L3 expression level was high but normal when expression was low. Olfactory memory was low (bold) when expression was low but was normal with high expression.

Finally, we have found that mutation of *aru* EPS8L3 can have specific effects in memory formation depending on the genetic background in which it is tested. Flies with the *aru^8–128^* insertion allele have altered place memory in the wild-type CS genetic background. The same *aru^8–128^* allele in the CS background does not alter the mutant flies' olfactory memory. In contrast, in the Berlin background the mutant flies have a significant defect in olfactory memory. It has been recently discovered that a region in the 3′ region of the *white* locus, which is often-times used in modified P-elements for mutagenesis, can act as a cryptic promoter [16]. When these modified P-elements are inserted upstream of a gene or within an intron, chimeric transcripts can be detected in which part of the *white* locus is fused with the gene that is closeby. It is plausible that the *aru^8–128^* insertion allele has a significant impact on *aru* EPS8L3 transcription to increase expression based on the cryptic promoter in this modified P-element. Thus, it is presumably the interaction of the Berlin and CS alleles at some number of genes with the *aru^8–128^* allele, and perhaps the cryptic promoter, that gives rise to either the over-expression or decreased expression of *aru* EPS8L3 and associated place and olfactory memory deficits. This genetic background-specific expression of a mutant phenotype is similar to the finding of mushroom body structural changes in flies mutant for one of several mushroom body development genes [17]. The results here provide the first example of a genetic background-specific effect of a mutation on memory formation in *Drosophila*.

## Materials and Methods

### Flies and rearing conditions

The Canton S (CS) and Berlin strains were used as wild-type flies. The *aru^8–128^*, *aru^S8^*, and *aru^S13^* flies were isolated as part of a screen for identifying genes important for regulating the effects of ethanol on behavior [4]. Flies with the *aru^8–128^* allele were introgressed for at least six generations with a *w^1118^* allele that has either been ‘Cantonized’ or ‘Berlinized’. Before behavioral experiments were carried-out the X-chromosome was replaced with a wild-type version to avoid measuring *white* mutant memory effects [18]–[21]. The *aru^S8^* and *aru^S13^* alleles were introgressed with the cantonized and berlinized *aru^8–128^* flies before the mutant chromosomes were collected and X-chromosome replaced by wild-type versions with balancer crosses. The flies were raised on cornmeal/yeast media at 24°C and 60% relative humidity on a 12 h L:D cycle. Flies were between 2 and 7 days of age and were never anesthetized for the behavioral experiments.

### Behavioral Experiments

Two types of learning experiments were carried out. These are the heat-box place learning and classical olfactory conditioning paradigms. Behavioral control experiments test the ability of flies to sense and avoid high temperatures, olfactory cues, and electric shock.

The heat-box was used for place conditioning. In this apparatus, single flies are allowed to walk in a chamber that is lined top and bottom with Peltier elements [22]. The position of the fly is detected by a bar code reader; a computer coordinates rising temperatures with position of the fly [23]. One half of the experiments associate high temperatures with the front half of the chamber. The other experiments associate high temperatures with the back half. Flies were allowed to walk in the chamber for 30 seconds during a pre-test phase. Conditioning followed the pre-test for twenty minutes with the aversive temperature set at 41°C. Place memory measured directly after training for three minutes provides a single measure of a memory with several components [24]–[27]. During the memory test the chamber temperature was kept constant at 24°C. A performance index for memory was calculated as the time in the punishment-associated chamber half subtracted from the time in the non-punishment-associated chamber half, all divided by the total time in a given training session [22]. The maximum performance index is 1.0 and indicates perfect avoidance of the chamber-half previously associated with high temperature. A performance index of zero indicates preference for neither chamber half.

We use a thermosensitivity assay to test for the ability of flies to sense and avoid a high temperature source [26], [28]. These tests use the same chambers; the difference is that the temperature of each chamber half is manipulated independently of fly behavior. Following one minute when both chamber halves are held at 24°C, one chamber half is warmed to 41°C for one minute. A performance index is calculated in the same fashion as in the learning experiment. An equal number of experiments start with the 41°C side in the front or back of the chamber.

Classical olfactory conditioning paired one of two odorants (4-methylcyclohexanol and 3-octanol) with electric shock (1.3 sec 100 volt shocks were applied every 5 sec for one min) [29]. The undiluted odorants were held in odorant cups, where air was passed over them into either the shock-tube or into the odor choice tubes in the memory test. Memory tests were performed 3 minutes after training for one min, where changed olfactory preferences were tested in a T-maze. The odorant associated with shock alternated between experiments. A performance index was calculated for the learning and control experiments and multiplied by 100, as is the tradition for this assay. This scale ranges from −100 to 100, with 0 indicating no memory or avoidance behavior. This was calculated as the number of flies choosing the shock-associated odorant subtracted from the non-shock-associated odorant, divided by the total number of flies in a ‘half-test’. An average PI was calculated from a pair of half-test PIs where each half came from conditioning of one of the two odorants.

Control experiments for classical olfactory conditioning measured flies' avoidance of the odors or shock used in the conditioning experiment. That is, odor at the same concentration used in the conditioning experiments was presented in one arm of the T-maze for one min. The other arm of the T-maze had air from the lab. In the shock test, two shock tubes were placed at the T-maze choice point and one of these was pulsed with 1.3 sec 100 volt electric shocks every 5 sec for one minute. The number of flies in both tubes were again counted to generate an avoidance performance index.

### Statistics

Place memory and thermosensitivity scores were tested using non-parametric statistics since tests for normality were rejected (not shown) [27], [30]. Two groups were compared using the Kolmgorov-Smirnov Test. When more than two groups were examined, multiple Kolmgorov-Smirnov Tests were performed with experimental genotypes against the control genotype. A Bonferroni correction was used to adjust the P-levels required to assign significant differences. Tests for significant differences in olfactory conditioning, control experiments, and mRNA levels used a parametric ANOVA with Neuwmann-Keuls post-hoc tests, when warranted [31]. Statistica software was used for all tests.

### Molecular Biology

The insertion site of the pGawB P-element in the *aru^8–128^* line was determined by inverse PCR and sequencing [32]. Inverse PCR followed genomic DNA restriction digest with HpaII, ligation, and PCR using primers GTC CGC ACA CAA CCT TTC C/GAG GAT GAC ATG TCG GAT GG or primers CGG GAC CAC CTT ATG TTA TTT C/CTG AGT GAG ACA GCG ATA TG. The sequenced PCR products were compared to the *Drosophila* genome to identify the P-element location. The insertion site was confirmed using PCR with primers annealing in the inverted repeats of the P-element (Primer 1 in Fig. 3: CGG GAC CAC CTT ATG TTA TTT C) and specific for the sequence on either side of the P-element in the adjacent genome (Primer 2 in Fig. 3: TCG CAC ATT ACT GTG AAG CCT; Primer 3 in Fig. 3: CCA TAA ACC TGG AGA CAT GC). After out-crossing the P-element, presence of the insertion was confirmed by PCR [4].

Quantification of *aru* EP8L3 expression levels was performed on mRNA extracts from fly heads. Approximately 200 fly heads were separated from bodies in liquid nitrogen. Total RNA was extracted using the Trizol reagent (Invitrogen, San Diego, CA). mRNA was purified with an mRNA mini-purification kit (Qiagen, Valencia, CA). 100 ng of mRNA was used as a template for cDNA synthesis from three or four independent extractions using a reverse transcriptase (Superscript III, Invitrogen). The *aru* EPS8L3 and *rp49* genes were used as templates for mRNA level quantification using the Applied Biosystems 7500 Fast Real-Time PCR system and their Power SYBR Green PCR master mix (Foster City, CA). The primers for *aru* EPS8L3 were: CGC CAT GGA GCT ATA CAA CA and TAT CAT CTT GCC GCT TCT CA. The primers for the *rp49* gene were: CCA GTC GGA TCG ATA TGC TA and GTT CGA TCC GTA ACC GAT GT. The efficiency of amplification was determined for each gene using a series of twofold cDNA dilutions, which were ultimately used in calculating relative concentrations of *aru* EPS8L3 transcript [33]. The efficiencies for the *aru* EPS8L3 and *rp49* genes were 1.99 and 1.98, respectively.
